# Measuring the Organizational Change Maturity of Chilean Companies

**DOI:** 10.3389/fpsyg.2021.791106

**Published:** 2021-12-17

**Authors:** Pablo Mac Carte, Paula Fariña

**Affiliations:** Faculty of Engineering and Science, Universidad Diego Portales, Santiago, Chile

**Keywords:** change management, strategic change management (SCM) index, confirmatory second-order factor analysis, reliability, validity, Lidership, organizational culture

## Abstract

This research presents the Strategic Change Management index, an indicator measuring the level of maturity of organizations to address processes of organizational change. At present, there is no other available indicator that fulfills this function. The index is built using the information provided from an instrument (questionnaire) specially created for this purpose. The instrument was applied to a sample of 151 companies, mostly Chilean. Studies about reliability (Cronbach’s α, hierarchical ω coefficients, among others), and instrument validity (second-order confirmatory factor analysis and retrospective validity) are presented. These studies show that the instrument has good psychometric properties. The results show that the degree of maturity of the companies comprising the sample to face change processes is low: 87% of the companies have a basic, initial, or amateur level of maturity; 13% have a professional level; and only one company had an expert level. More validity studies are required. However, the absence of a similar available instrument restrains the realization of more in-depth validity studies at this time.

## Introduction

In this research, we present the Strategic Change Management (SCM) index, an indicator that measures the level of maturity of organizations to face processes of organizational change. To do so, we explore two sources of information: on one hand, we build on previous scientific literature: Marín-García et al. (2008), Cameron and Quinn (2011), Chacón (2013), Correa (2017), Hussain et al. (2018), and Valencia-Rodríguez (2019). On the other hand, we nurture the SCM model with public information provided by Professional Change Management Organizations such as Prosci and Accelerating Implementation Methodology [AIM] (2019).

Several authors postulate that organizations need to develop adaptability capacities to survive environmental changes (Rodriguez, 2011; Alvesson and Sveningsson, 2015; Hussain et al., 2018). Change management (CM) is a management practice to guide processes of internal change in organizations. It is especially focused on the role played by the stakeholders of the change project when implementing and sustaining the required change over time. Likewise, CM seeks to reduce the resistance to change that occurs in processes of organizational transformation, increasing the degree of commitment of stakeholders to better face the three phases of Lewin change projects: the unfreezing, changing, and refreezing (see Lewin, 1947).

Adapting to the environment needs the development of innovating capacities; it also requires an inspiring leadership style and the conformation of high-performance work teams. Rodríguez (2001) also includes the need of designing business models capable of growing exponentially, and of installing dynamic and flexible organizations.

**FIGURE 1:**
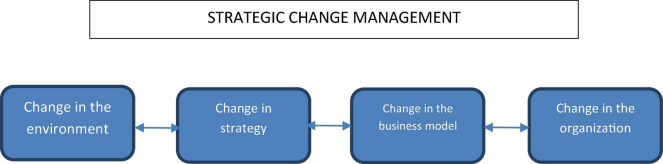
Diagram on the new way of understanding strategic change management (SCM).

Figure 1 shows our strategic view of organizational change. The two-way relationship between environment, strategy, business model, and organization assumes that the organizational change must be in homeostasis with the external factors that motivate the change. Our model also requires a strategic vision of change. Our ideas are in line with contemporary authors such as Bridges (2019), Nadler and Tushman (2019), and others.

The aim of the SCM indicator is that organizations identify their state of maturity for managing change and thus anticipates the chances of success of their strategic change projects. It measures five stages of change maturity, namely, (1) *Basic stage*: organizations that are unaware of the real impact of CM and have basic notions about its methodology; (2) *Initial stage*: organizations that are aware of the impact of CM, but have neither formal training nor understand distinctions; (3) *Amateur stage*: perform CM processes without a formally trained team, but they are self-taught, therefore, inefficient and ineffective; (4) *Professional stage*: CM is part of the organizational strategy and culture, and they continuously invest in staff training; and (5) *Expert stage*: experimentation reaches its optimum and they begin to innovate in CM processes, maximizing flexibility, agility, collaboration, and outcomes.

In turn, the indicator identifies four dimensions to manage organizational change: Leadership, Culture, Continuous Improvement, and Capabilities. This indicator allows organizations to ascertain their gaps in relation to each dimension so that they can efficiently invest resources to enhance their capacities to manage change.

**FIGURE 2:**
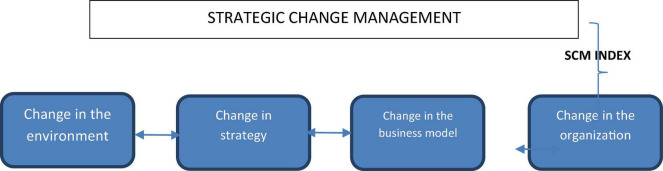
Diagram on the scope of the SCM indicator within the organizational change process.

The SCM indicator should be used in the organizational change phase to provide clarity on the level of preparation of the business to address strategic transformation processes. Organizations can use this information to identify whether their current stage will promote or hinder the desired change. In turn, this will help to anticipate whether it is convenient to start such a transformation process that could be negatively affected if the organization does not possess the internal capacities to avoid all the organizational and individual difficulties and barriers related to change. Figure 2 shows the scope of the SCM indicator within this process.

### Background of the Strategic Change Management Model

Previous models that reflect the state of CM in organizations were analyzed to design, contrast, and add value to the SCM model. *Prosci’s Change Management Model* considers five dimensions: leadership, application, competence, standardization, and socialization, see Prosci (2020a). It evaluates 50 specific, observable traits across the five capability areas, using a rubric scoring system. Each of the 50 traits is presented to deployment leaders with descriptions of an organizational level (from 1 to 5). The users must select the description that best matches the organization (Prosci, 2020b).

*The Organizational Maturity Model for Change Management* (Chacón, 2013) establishes five degrees of organizational maturity to manage change in companies. The model design includes the identification of best practices related to the individual, programs and projects, and organizational dimensions of the change processes, and the best practices related to definition/standardization measurement control, and improvement processes. This model bases its theory on the fact that changes must be developed in compliance with quality standards so that their management can incorporate tools associated with quality management. This model measures change management maturity by identifying the level of best practices used to manage change at the individual, project, and organizational levels. For each practice, it measures its degree of definition, measurement, control, and improvement. The instrument used to measure best practices has 140 questions. The level of maturity is expressed with an index ranging from 0 to 100%, from very low to high maturity. Change management is measured at levels, namely, individual, project, and organizational levels.

*The Maturity Levels of Capabilities to Manage Change* (Correa, 2017) establishes five degrees of organizational maturity to manage change in companies. As in Prosci’s model, in this model, the companies determine their level of maturity according to the definitions of the stages, without generating an index. In fact, the definition of each level is very similar to that proposed by Prosci. Finally, *the Change Diagnostic Index model* (Osipova and Ayupova, 2013) helps to measure organizational resistance to change before, during, and after a change initiative. Unlike the other models above, this model does not directly measure the level of maturity to address the change process. It measures the resistance (at the individual and organizational levels) experienced by an organization during a change process. It also proposes strategies and tactics to manage this resistance.

The contribution of this research is to offer the SCM index, a tool to measure the level of maturity of an organization to address processes of organizational change.

## Materials and Methods

### Strategic Change Management Model

**FIGURE 3:**
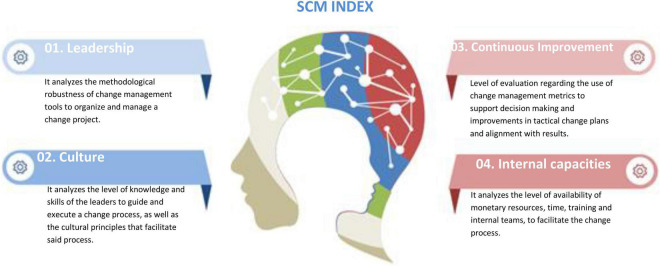
Explanatory diagram of the SCM Index with its four dimensions.

**FIGURE 4:**
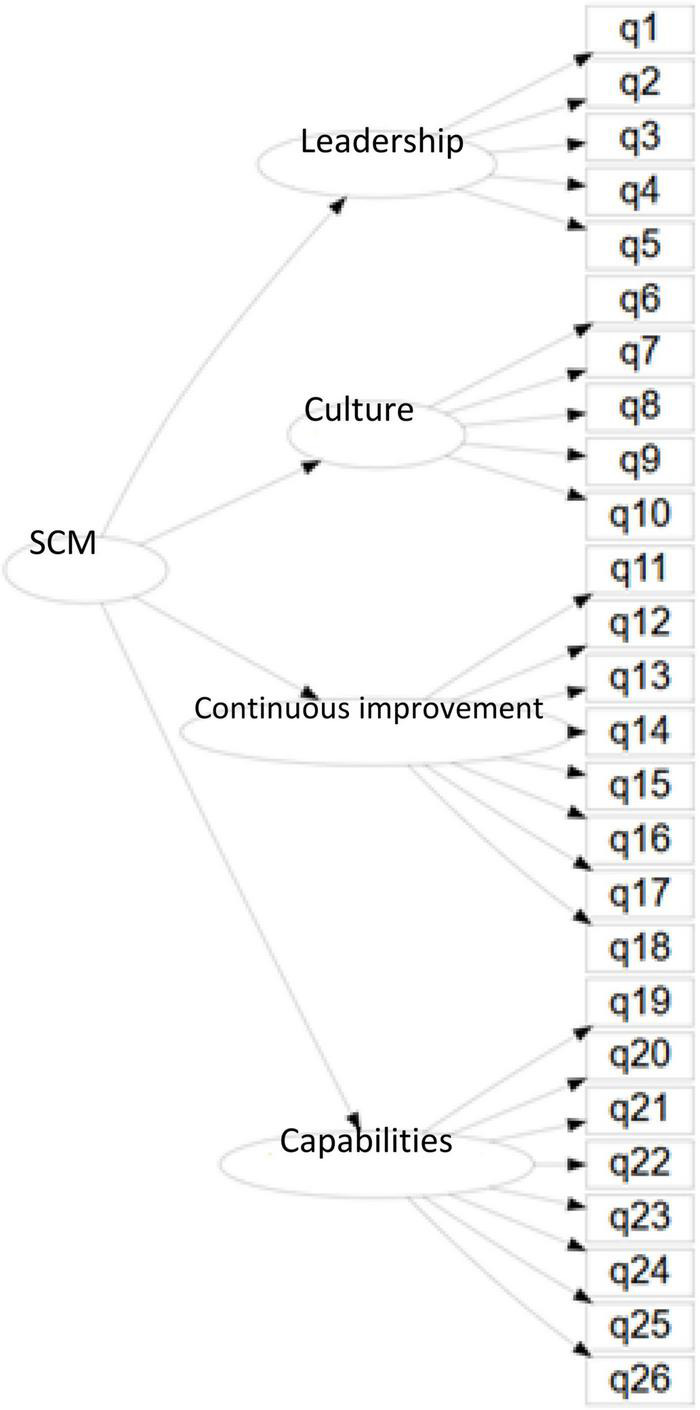
Strategic change management model diagram.

Building on the previous literature (section “Background of the Strategic Change Management Model”), we propose the SCM model. The new model considers four dimensions, namely, Leadership, Culture, Continuous Improvement, and Internal Capacity to explain the level of maturity of organizations to face processes of organizational change (see Figures 3, 4). The SCM model is coherent with the theoretical previous literature summarized below.

#### Culture

Previous authors have considered the importance of the organizational culture for a successful change process (Cameron and Ettington, 1988; Kotter and Heskett, 1992; Gross et al., 1993; Caldwell, 1994; CSC Index, 1994; O’Reilly and Chatman, 1996; Cameron and Quinn, 2011). According to these authors, culture works as a social glue within organizations, and, therefore, it is relevant to diagnose the ability of these organizations to adapt to environmental changes.

#### Leadership

Regarding the leadership dimension, many authors affirm that leadership is vital during an organizational change process (Bass, 1985; Higgins et al., 2003; Hussain et al., 2018). A leader who encourages their workers will gain their support and feedback, which are essential aspects of a successful change process. In fact, the definition of leadership itself—a process by which an individual influences a group of individuals to achieve a common goal (Northouse, 2004)—underlines the importance of leadership during an organizational change process.

#### Continuous Improvement

Following Marín-García et al. (2008), organizations that incorporate continuous improvement practices have better chances of adapting to new environments. Continuous improvement is defined by Grütter et al. (2002) as small incremental changes in the productive processes or work practices that allow improving some performance indicator. Terziovski and Sohal (2000) add that continuous improvement does not require a large amount of investment to be launched, but do remark the necessity of involving the whole organization in the change process. Continuous improvement has evolved from the four-step Deming cycle (Langley et al., 2009)—Plan, Make, Verify, and Act—to the Lean (Womack et al., 1990) and Six Sigma methodological models. These models are used to improve productive lines, organizational processes, and products.

#### Internal Capacity

Moran and Brightman (2001) incorporate the idea of capacity when they define change management as the process of continuous renewal of the direction, structure, and capacities of an organization to attend to the changes needed by external and internal clients. The internal capacity has also been pointed out as an important aspect to afford a change in the organizations by Valencia-Rodríguez (2019).

Table 1 compares our model with the other three models employed by Professional Change Management Organizations, namely, Standard for Change Management of ACMP (Association of Change Management Professionals [ACMP], 2019), Prosci Change Management, AIM Change Management, and HUCMI Change Management (HUCMI, 2019). It can be noticed that our proposed four dimensions are coherent with them.

**TABLE 1:** Comparison between the strategic change management (SCM) model and other Professional Change Management Organization models.

Models	SCM	ACMP	Prosci	AIM	HUCMI
Change model	X	X	X	X	X
Maturity indicator	X		X		X
*Culture*		X	X	X	X
*Leadership*		X	X	X	X
*Continuous improvement*		X		X	
*Internal capacity*		X	X	X	

*SCM, strategy change management; ACMP, standard for change management of ACMP; AIM, AIM change management; HUCMI, HUCMI change management.*

### The Questionnaire

We created an instrument (questionnaire) composed of 26 Likert-type questions with five levels. The questions are presented in Table 2. They are divided into the four SCM dimensions, namely, Leadership (L) with five questions, Culture (CU) with five questions, Continuous Improvement (CI) with eight questions, and Capabilities (CA) with eight questions.

**TABLE 2:** Second-order confirmatory factor analysis (CFA) model results and description of SCM questionnaire items.

	Weights (s.e.)	Questions of the SCM questionnaire
**First order: SCM index**		
Leadership	2.393 (0.419)	Latent construct
Culture	2.186 (0.505)	Latent construct
Continuous improvement	1.237 (0.182)	Latent construct
Capabilities	2.683 (0.480)	Latent construct
**Second order: sub-indexes**		
**Leadership**		
Question 4	0.404 (0.067)	Have the change projects been sponsored?
Question 5	0.457 (0.068)	Have the sponsors of change projects participated in communicating and motivating those involved?
Question 6	0.373 (0.058)	Has the person responsible for leading the change process managed resources and/or collaboration from other areas or units of the company?
Question 7	0.284 (0.060)	How often do change leaders meet with project teams?
Question 10	0.374 (0.057)	Do those responsible for leading change identify and train project team members to become agents of change?
**Culture**		
Question 8	0.384 (0.075)	How often do those leading the change process form multi-department teams to manage change projects?
Question 9	0.342 (0.068)	Do change leaders give responsibilities and autonomy to their teams?
Question 11	0.350 (0.071)	Are historically known cases of successful change projects systematically recognized to promote a culture of organizational change?
Question 12	0.374 (0.074)	Are there any pilot experiences to manage change?
Question 13	0.392 (0.079)	During the change process, is experimenting encouraged to learn from successes and mistakes?
**Continuous Improvement**		
Question 3	0.484 (0.066)	Do your project change management plans have a Vision and Mission?
Question 15	0.571 (0.075)	How often is internal client satisfaction verified in change projects?
Question 23	0.778 (0.078)	Is the level of adoption of new working practices by the people involved in the transformation projects measured?
Question 24	0.656 (0.087)	Is the speed of adoption of new working practices by the people involved in the transformation projects measured?
Question 25	0.830 (0.084)	Is the level of alignment between the people involved in the transformation process and the objectives and goals of the change projects measured?
Question 26	0.836 (0.084)	Is the degree of motivation of the people involved in the change processes measured?
Question 27	0.710 (0.070)	Does your company incorporate Change Management indicators as a relevant input in the decision-making process of transformation projects?
Question 28	0.497 (0.104)	Is the return on investment of the Change Management plan initiatives measured?
**Internal Capacity**		
Question 1	0.463 (0.078)	Does your company incorporate the concept of Change Management as a strategy support function?
Question 14	0.380 (0.059)	How often does your organization use Change Management methodologies in transformation projects?
Question 16	0.370 (0.061)	Are structured and formal communication plans developed in change projects?
Question 18	0.350 (0.061)	Do change projects have budgets for their correct execution?
Question 19	0.343 (0.061)	Does the organization assign a budget for training (courses, diplomas, workshops, others) in Change Management?
Question 20	0.368 (0.059)	Does the organization assign specialized Change Management teams in the change projects?
Question 21	0.324 (0.070)	Does the company have professionals trained in Change Management?
Question 22	0.350 (0.061)	Does the company set goals and objectives linked to Change Management in transformation projects?
*N*	151	

*Values in parentheses are standard errors.*

A group of six professionals (statisticians and change management experts) was called to design the survey. A pilot survey was applied to corroborate the correct understanding of the questions.

The instrument is used to both (1) corroborate the correctness of the SCM model through a second-order confirmatory factor analysis, and (2) measure the four sub-indexes (one for each dimension) and the global change management index (SCM) we propose.

### The Sample

A web form of our instrument was sent to 35,000 people by email. The information contained in the emails was provided by the Faculty of Economics and Business of the Diego Portales University. It includes the whole dataset of graduates and candidates from the graduate programs of this institution. The rate of non-responses was high, mainly because the surveys did not reach the recipient properly (the database was not updated). Fortunately, each contacted person belonged to different organizations. This was important since our interest was focused on the state of change management maturity of the organizations, not on the individuals.

The final sample includes 151 companies (unit sample), being 96% of them Chilean organizations of various areas and sizes. Even though the feasible sampling process cannot assure a representative sample from the Chilean firms, the sample can be useful to evaluate the proposed questionnaire and indexes.

**FIGURE 5:**
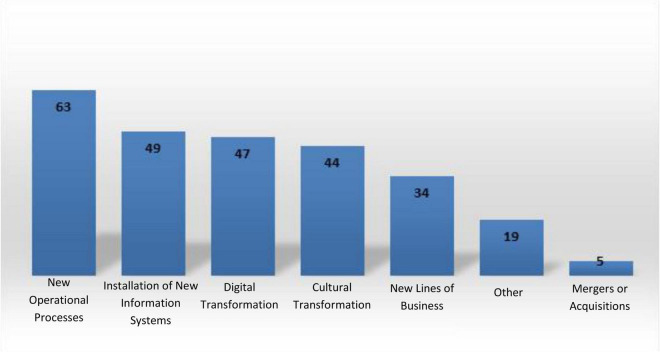
Frequency of the types of processes of changes within the companies comprising the sample.

In each company, the person leading to change projects was interviewed, assuming they were the person who best understood change management within their institutions. Respondents held various positions such as sponsor, change lead or agent, project manager, coach, and so on. These interviewees had participated in 2.8 internal change projects on average, with operational change projects being the most frequent ones (see Figure 5 for more details). The age of the respondents goes from 35 to 38 years, and 70% of them have professions related to business management. Most of these professionals are commercial and industrial engineers or auditing accountants. The remaining 30% of the respondents come from other areas of engineering, social sciences, communications, and health sciences.

### The Strategic Change Management Index and Sub-Indexes

Sub-indexes are obtained by the average score of the questions associated with each dimension, while the overall index is a weighted average of the sub-indexes. The weights assigned to each sub-index to build the global index were determined based on the most expert participants in change management of the sample: the expert and/or professional respondents considered L, CU, CA, and CI to be very or highly important, scoring 85, 80, 60, and 55%, respectively. Following this order, we assign 0.3 for the L dimension; 0.27 for CU; 0.23 for CA; and 0.20 for CI. Equations 1–5 formally define the sub-indexes and global index, respectively.

(1) $$L=\frac{1}{5}\,\left(Q\,4+Q\,5+Q\,6+Q\,7+Q\,10\right)$$

(2) $$C\,U=\frac{1}{5}\,\left(Q\,8+Q\,9+Q\,11+Q\,12+Q\,13\right)$$

(3) $$\;\,C\,A=\frac{1}{8}\,\left(Q\,1+Q\,14+Q\,16+Q\,18+Q\,19+Q\,20+Q\,21+Q\,22\right)$$

(4) $$C\,I=\frac{1}{8}\,\left(Q\,3+Q\,15+Q\,23+Q\,24+Q\,25+Q\,26+Q\,27+Q\,28\right)$$

(5) S⁢C⁢M=0.3⁢L+0.27⁢C⁢U+0.2⁢C⁢I+0.23⁢C⁢A

Since each question takes integer values between 1 and 5, the index and sub-indexes also move between 1 and 5. The higher the value of the indexes, the better the degree of maturity. Companies are classified in the five stages of maturity described in the “Introduction” section of this article are as follows: *basic* if the index/sub-index is in the range [1,2), *initial* when the index/sub-index is in the range [2,3); *amateur* for the range [3,4); *professional* for the range [4,5); and *expert* only if a 5 is obtained for the corresponding index/sub-index.

### Assessment Procedures

We propose a Second-order Confirmatory Factor Analysis measurement model (Second-factor CFA) to assess our theoretical SCM model. Figure 4 illustrates that the Second-factor CFA is a proper measurement model.

First, an exploratory factor analysis (EFA) is employed to corroborate the hypothesized model structure. We corroborate the number of factors of the structural model as well as the relevant questions for each construct, without prior assumptions about the model.

Second, we employ reliability indexes to analyze internal consistency. Cronbach’s α, Cronbach (1951), Guttman’s λ3 and λ6, Guttman (1945) coefficients are appropriate for analyzing the reliability of the sub-indexes (Eqs 1–4), since their use requires that the questions measure a single latent construct. They present a lower limit to reliability (Guttman, 1945; Lord and Novic, 1968). The correlation between each sub-index and the SCM index (Eqs 1–4 vs. 5) and the correlation index proposed by Lord and Novic (1968), Equation 4.7.4 is also used. As stated in previous literature (see Zinbarg et al., 2005; Cho, 2016; Trizano-Hermosilla et al., 2021), the multidimensional structure of a test or index requires specific reliability coefficients. When the questions that make up the index have a multidimensional structure, Cronbach’s coefficient can deliver a poor lower limit for reliability. Instead, it is proposed to use the ω-hierarchical coefficient and the reliability coefficient for the second-order factor models proposed by Cho (2016) to measure the reliability of the global index (Eq. 5). Since we do not have a benchmark instrument available, external consistency was not possible.

Third, convergent validity is analyzed through the significance (5%) of the loading coefficients, and the use of model fit tests and indicators. Specifically, we employ the Confirmatory Factor index (CFI), the Tucker Lewis index (TLI), the Root Mean Square Error of Approximation (RMSEA), and the chi-square test. All the indicators and tests are calculated in the standard version (assuming normality) and the robust version (relaxing normal assumptions).

The CFI assesses the ratio of the deviation of the proposed model from the null model (without correlations) against the deviation of the saturated model from the null model. It moves from 0 (the worst fit) to 1 (a better fit). The TLI is the ratio of the deviation of the null model from the proposed model to the deviation of the null model to the saturated model. The more similar the deviation from the null model is, the closer the ratio to one is. A TLI indicator smaller than 2 indicates a good fit (Marsh and Hocevar, 1985).

The RMSEA is an absolute measure of fit that compares the chi-squared statistic of the proposed model with its theoretical degree of freedom (theoretical mean). Values under 0.08 are considered a reasonable fit, and smaller than 0.05 a close fit. We also include the LR chi-squared test that contrasts the null hypothesis that the null model is better than the proposed model. Nevertheless, it is well documented (Tucker and Lewis, 1973; Bentler and Bonett, 1980; Sharma et al., 2005) that this test used to be biased (always rejected with sample sizes bigger than 100).

Finally, to give an additional element to the validity analysis, the satisfaction survey report is used in view of the results of the implementation of organizational changes in the company. Specifically, respondents were asked about their level of satisfaction with the achievement of project objectives, meetings established timelines, and the assigned budget, adherence to the required cultural change, and organizational commitment to change. All this is in reference to organizational change processes prior to sampling. We believe that this variable can shed light on the effectiveness of the SCM index. Indeed, it is likely that high levels of satisfaction in organizational change processes already carried out are due to the company’s high level of maturity in implementing this type of change. This should also be reflected in a high SCM index.

## Results

### Descriptive Statistics for the Strategic Change Management Dataset

Tables 3, 4 show descriptive statistics of the companies of the sample and of the sub-indexes and index, respectively. The application of the survey suggests that the degree of maturity of companies to address change processes is low: 87% of them had a basic, initial, or amateur level; 13% were professional; and only one company reached the expert level. The dimension in which companies were best evaluated was leadership (sub-index average is 3.2), followed by culture and capabilities. The Continuous Improvement dimension seems to be the weakest aspect of the companies of the sample, with a sub-index average of 2.2. The Pearson correlations between the sub-indexes are high (between 0.79 and 0.58), with the Continuous Improvement sub-index having the lowest correlations with the remaining sub-indexes (between 0.58 and 0.70).

**TABLE 3:** Descriptive statistics of the companies of the sample.

Activity	No. of companies	Percentage
Trade	5	4.35
Construction	5	4.35
Education	15	13.04
Industry	9	7.83
Mining	2	1.74
Other	19	16.52
Health	9	7.83
Public sector	11	9.57
Services	40	34.78
Small businesses (0–10 employees)	20	18
Small companies (11–50 employees)	30	27
Medium-sized companies (51–250 employees)	17	15
Large companies (250 employees or more)	44	40

**TABLE 4:** Descriptive statistics of the sub-indexes and index.

	Leadership	Culture	Continuous	Capabilities	SCM
			Improvement		
Minimum	1.0	1.0	1.0	1.0	1.0
First quartile	2.4	2.4	1.3	2.0	2.2
Mean	3.2	3.0	2.2	2.9	2.9
Third quartile	4.1	3.8	2.9	3.9	3.6
Maximum	5.0	5.0	5.0	5.0	5.0
Basic	11.92	15.23	50.99	24.50	15.89
Initial	28.48	31.13	23.84	24.50	41.06
Amateur	27.81	34.44	12.58	27.81	29.80
Professional	29.14	17.22	10.60	20.53	12.58
Expert	2.65	1.99	1.99	2.65	0.66
Leadership		0.79	0.58	0.72	0.90
Culture			0.58	0.70	0.88
Continuous Imp.				0.70	0.80
Capabilities					0.89

*The rows from 11 to 14 present sub-indexes and index Pearson’s correlation matrix.*

### Exploratory Factor Analysis

Table 5 presents eigen-values of the 26 questions’ variance–covariance matrix. Four values are bigger than one, suggesting a four-factor model.

**TABLE 5:** First line: the biggest six eigenvalues of Pearson variance–covariance matrix of observable variables (questions).

Eigenvalues	12.30	1.97	1. 26	1.02	0.91	0.84

	**EFA Model 1**	**EFA Model 2**	**EFA Model 3**	**EFA Model 4**	**EFA Model 5**	**EFA Model 6**
χ^2^ test: stat-df (*p*-value)	733.72–299(1. 17e-38)	471.49–274(1.19e-12)	393.49–225(1.75e-8)	302.44–227(5.96e-4)	244.68–205(0.03)	202.67–184(0.164)

*Second line: chi-squared test which null hypothesis is “the number of factors is sufficient.”*

Table 6 presents the results of the four-factor EFA model. Each factor relates to one of the SCM model’s dimensions. Factor 1 presents high values of the loadings for the questions related to the capacity dimension, factor 2 is linked with Continuous Improvement, and factors 3 and 4 are related to Culture and Leadership, respectively. Only the question loadings 3, 7, and 10 are not bigger than the remaining loadings of the same question at different factors.

**TABLE 6:** Four-factors exploratory factor analysis.

Loadings	Factor 1 (capacity)	Factor 2 (continuous improvement)	Factor 3 (culture)	Factor 4 (leadership)	Uniqueness
Question 1	**0.539**	0.267	0.236	0.209	0.538
Question 3	0.401	**0.346**	0.324	0.134	0.596
Question 4	0.394	0.109	0.438	**0.549**	0.339
Question 5	0.409	0.239	0.508	**0.716**	0.005
Question 6	0.483	0.248	0.499	**0.323**	0.352
Question 7		0.175	0.458	**0.219**	0.710
Question 8	0.424		**0.680**	0.147	0.333
Question 9	0.354	0.183	**0.606**	0.119	0.459
Question 10	0.419	0.287	0.565	**0.297**	0.334
Question 11	0.267	0.275	**0.571**	0.181	0.494
Question 12	0.268	0.229	**0.683**		0.406
Question 13	0.228	0.349	**0.654**	0.195	0.360
Question 14	**0.639**	0.419	0.360	0.257	0.221
Question 15	0.249	**0.590**	0.228		0.536
Question 16	**0.665**	0.291	0.383	0.137	0.308
Question 18	**0.508**	0.261	0.332	0.125	0.547
Question 19	**0.625**	0.214	0.174		0.531
Question 20	**0.638**	0.273	0.196	0.212	0.435
Question 21	**0.382**	0.240	0.147		0.765
Question 22	**0.520**	0.330	0.318		0.517
Question 23	0.264	**0.738**	0.248		0.316
Question 24	0.291	**0.666**	0.173		0.442
Question 25	0.294	**0.778**	0.250		0.240
Question 26	0.237	**0.779**	0.187	0.186	0.268
Question 27	0.453	**0.528**	0.320		0.413
Question 28		**0.450**		0.134	0.768
SS loadings	4.583	4.366	4.363	1.454	
Proportion Var	0.176	0.168	0.168	0.056	
Cumulative Var	0.176	0.344	0.512	0.568	

*The values in bold are the questions included in each SCM sub-index.*

### Consistency

Table 7 shows several reliability coefficients.

**TABLE 7:** Reliability indexes.

	Leadership	Culture	Continuous improvement	Capabilities	SCM
Cronbach α	0.86 (0.84–0.9)	0.86 (0.83–0.9)	0.89 (0.86–0.92)	0.87 (0.84–0.9)	
Guttman λ_3_	0.87	0.88	0.88	0.86	
Guttman λ_6_	0.87	0.84	0.89	0.89	
Cor (SCM-Subindex)	0.87	0.86	0.85	0.92	
ω-hierarchical					0.88
Second order coefficient					0.94

*95% confidence bounds between parenthesis.*

Cronbach’s α, Guttman’s λ3 and λ6 coefficients are appropriate for analyzing the reliability of the sub-indexes (see section “The Sample”). High reliability is observed for all four sub-indexes. Pearson’s correlations between the sub-indexes are high (between 0.79 and 0.58). Besides, the correlation between each sub-index and the SCM index shows that the four sub-indexes have a high correlation with the SCM index. Almost all the question/sub-indexes-without-this-question correlations are above 0.6. The lowest value was 0.44 for a question on the Capabilities sub-index (see Table 8).

**TABLE 8:** Question-sub-index without this question correlation.

Leadership	Culture	Continuous improvement	Capacity
Q4: 0.72	Q8: 0.77	Q3: 0.56	Q1: 0.60
Q5: 0.85	Q9: 0.73	Q15: 0.67	Q14: 0.80
Q6: 0.74	Q11: 0.68	Q23: 0.81	Q16: 0.74
Q7: 0.46	Q12: 0.73	Q24: 0.74	Q18: 0.61
Q10: 0.71	Q13: 0.76	Q25: 0.85	Q19: 0.63
		Q26: 0.83	Q20: 0.70
		Q27: 0.73	Q21: 0.44
		Q28: 0.49	Q22: 0.63

Table 7 also shows that the reliability indicators for the global index: the ω-hierarchical coefficient and the reliability coefficient for the second-order factor models proposed by Cho (2016) yield high levels of reliability. Finally, the question-index correlations with the omitted question have an average of 0.65. The minimum value is 0.37 and the maximum value is 0.83.

### Validity

The lavaan library of R (Rosseel, 2012) was used to estimate the Second Order-factor CFA model by maximum likelihood. The process involved estimating 56 parameters (30 loads and 26 variances). The factor variances were set to 1 for identification purposes.

All, first- and second-order loadings were significant at 5% of significance, reaffirming the correct specification of the model (see Table 2 for details of the second-order CFA model estimation). Table 9 shows the indicators of model fit: the CFI is close to 1; the TLI is smaller than 2; and the RMSEA is smaller than 0.08. In other words: the three indicators show a good fit of the model. Contrary, the LR chi-squared test rejects the null hypothesis that the model adjustment is proper. We conclude that convergent validity is corroborated since the model fits properly [it has been widely reported that the LR test has an excessive tendency to reject the null hypothesis (Tucker and Lewis, 1973; Bentler and Bonett, 1980; Sharma et al., 2005)].

**TABLE 9:** Fit measures of the proposed CFA model.

Test/indicator	Standard statistic/index (*p*-value)	Robust statistic/index value (*p*-value)
L ratio Chi squared	506.109 (0.000)	444.493 (0.000)
Model Test Baseline Model	2,808 (0.00)	2,909.426 (0.000)
Comparative fit index (CFI)	0.915	0.932
Tucker–Lewis index (TLI)	0.906	0.925
RMSEA	0.069 *CI*_90_ = (0.059, 0.079)	0.058 *CI*_90_ = (0.047, 0.068)

### Additional Element of Validity

Table 10 shows the relative frequencies of the SCM index ranges for each level of satisfaction. The five aspects of satisfaction reported (achievement of objectives, meeting timelines, compliance with budget, adherence to change, and organizational commitment to change) are considered.

**TABLE 10:** Relationship between SCM maturity levels and satisfaction levels of change processes within the company before the survey.

Level of satisfaction		Basic	Initial	Amateur	Professional	Expert
Achievement	Very unsatisfied	58.33	33.33	8.33	0	0
	Unsatisfied	71.43	0	14.29	14.29	0
	Indifferent	47.22	30.56	11.11	11.11	0
	Satisfied	16.67	16.67	0	50	16.67
	Very satisfied	100	0	0	0	0
Timelines	Very unsatisfied	64.29	21.43	7.14	7.14	0
	Unsatisfied	80	0	20	0	0
	Indifferent	47.22	30.56	11.11	11.11	0
	Satisfied	0	20	0	60	20
	Very satisfied	80	20	0	0	0
Budget	Very unsatisfied	62.5	25	0	12.5	0
	Unsatisfied	85.71	0	14.29	0	0
	Indifferent	47.22	30.56	11.11	11.11	0
	Satisfied	16.67	16.67	0	50	16.67
	Very satisfied	62.5	25	12.5	0	0
Adherence	Very unsatisfied	75	16.67	8.33	0	0
	Unsatisfied	75	0	12.5	12.5	0
	Indifferent	47.22	30.56	11.11	11.11	0
	Satisfied	0	20	0	60	20
	Very satisfied	50	50	0	0	0
Commitment	Very unsatisfied	70	30	0	0	0
	Unsatisfied	71.43	0	14.29	14.29	0
	Indifferent	47.22	30.56	11.11	11.11	0
	Satisfied	0	20	0	60	20
	Very satisfied	71.43	14.29	14.29	0	0

In the achievement of objectives aspect, of the total number of people who declared to be *Very Satisfied* with this item (first block of the table), 66.7% obtained a professional or expert level in the SCM index. Of the people who claimed to be *Indifferent*, this figure drops to 11.1% of cases. Finally, none of the people who indicated to be *Very Dissatisfied* coincides with the professional range of the SCM index. This pattern is observed for the five aspects of satisfaction considered.

Accordingly, the levels of satisfaction with change processes prior to sampling are consistent with the measurements suggested by the SCM index. While this may provide arguments in favor of the validity of the instrument, it is important to clarify that this comparison has limitations, namely, (1) it is a subjective evaluation of the respondent, and (2) the satisfaction survey report is made by the same person who answered the questions that make up the SCM index. More research is needed to complement the validity study. The absence of a benchmark prevents a more in-depth study.

## Discussion

The SCM index helps organizations to determine their stage of maturity to manage change. With this tool, organizations will be able to determine in which stage they are, specifically regarding the gap that is restraining them to evolve in a programmed and planned manner. Measuring the stage of maturity enables the organization to identify a probability of occurrence of a stage of satisfaction in variables such as achievement of project objectives, meeting timelines, compliance with budgets of a project, and the degree of commitment and alignment of employees with the change process. As the organization’s maturity moves forward, the probability of greater satisfaction increases. Therefore, measuring the SCM index allows the organization to determine in a very simple manner if its projects will fulfill or not their expectations.

The SCM index has several advantages over previously proposed approaches. Unlike Prosci’s survey (Prosci, 2016), in the SCM survey, it is not the respondent who defines the level of their organization. In SCM, the respondent is blind to the result. They only find out after they have answered the entire questionnaire and their answers are processed. This makes the measurement more objective as it makes the respondent less likely to be biased toward an answer to achieve a desired but incorrect stage. Furthermore, SCM presents a continuous result through its global indicator (a percentage) and its four dimensions, while Prosci’s method only presents a discrete result (five levels). Prosci (2016) reports frequencies of its indicators for Latin America of 24, 28, 38, 10, and 0% at levels 1–5, respectively. The SCM index shows for Chile 15.9, 41.1, 29.8, 12.6, and 0.6% at levels 1–5, respectively. These results must not be interpreted as an advantage of Chile with respect to the rest of Latin America since (1) the measures come from different indexes, and (2) our sample is not representative of the Chilean organizations. Nevertheless, it shows consistency between Prosci’s and SCM indexes.

With respect to the differences with the model proposed by Chacón (2013), the SCM index considers leadership as a critical dimension for managing change in organizations. Even though Chacón establishes four dimensions, that is, strategy, structure, policies, and processes and culture, he analyzes leadership from a general function and not from how it should be performed to manage change. Unlike the form proposed by Chacón (2013), the SCM index makes differences in the roles that sponsors should play in the change processes. Moreover, Chacón places a strong emphasis on the documentation and standardization of change processes, elements that for the SCM index are not relevant, especially in the age we live in. The time and resources involved in documentation and standardization in constantly changing organizations restrain their ability to adapt. In this sense, the SCM index promotes agility over excessive standardization of documents. Another differentiating aspect of the SCM index is that it considers the capacity for innovation in change management tools as a relevant variable to evolve in the stage of maturity.

This study shows that the SCM index has suitable psychometric properties in terms of consistency and validity. However, further research is required to confirm the validity of the instrument. There are two lines of research that would be relevant to explore, namely, the predictive validity of the index and the application of the instrument to larger and representative samples. Denison et al. (2014) present a literature review on studies aimed at validating instruments that measure organizational culture. Although Denison focuses on instruments that measure aspects different from change maturity, the article remarks that it takes a long time to validate a measurement instrument that is intended to be used in contexts other than Chile.

The SCM index is a novel contribution to the battery of existing tools to study the level of maturity to manage change in organizations. It would be of great value to extend this analysis to measure the behavior of the SCM index in different countries or continents to determine in which countries the companies are better equipped to address change. It would also be interesting to study the correlation between the SCM index and the cultural elements and/or organizational leadership styles. Finally, it would be interesting to verify if the industries with a higher stage of maturity are within more uncertain and dynamic environments.

## Data Availability Statement

The raw data supporting the conclusion of this article will be made available by the authors, without undue reservation.

## Author Contributions

PM developed and applied the instrument. PF worked in the data analysis. PM and PF worked to wrote the manuscript and approved the submitted version.

## Conflict of Interest

The authors declare that the research was conducted in the absence of any commercial or financial relationships that could be construed as a potential conflict of interest.

## Publisher’s Note

All claims expressed in this article are solely those of the authors and do not necessarily represent those of their affiliated organizations, or those of the publisher, the editors and the reviewers. Any product that may be evaluated in this article, or claim that may be made by its manufacturer, is not guaranteed or endorsed by the publisher.
